# Biochemical Evaluation of Silver Nanoparticles in Wistar Rats

**DOI:** 10.1155/2014/196091

**Published:** 2014-10-30

**Authors:** Oluyomi Stephen Adeyemi, Ifeoluwa Adewumi

**Affiliations:** ^1^Department of Biological Sciences, Landmark University, Omu-Aran, Kwara State 370102, Nigeria; ^2^Department of Chemical Sciences, Redeemer's University, Redemption Camp, Mowe 121001, Nigeria

## Abstract

*Background*. Silver nanoparticles have found wider and increasing biomedical applications due to their broad antimicrobial characteristics. However, toxicity of nanoparticles is a subject of continued controversy, thus necessitating further studies in this direction. *Objectives*. This study investigated the biochemical effects of silver nanoparticles in Wistar rats. *Materials and Methods*. Forty male rats were randomly distributed into eight experimental groups of five. Group A served as the control and received distilled water. Groups B to H were orally exposed to varying concentrations of silver nanoparticles (AgNPs) at 100, 1000, and 5000 mg/kg daily for 7, 14, and 21 days alternately. Following cessation of treatments, rats were sacrificed and the blood and other vital organs were collected and prepared as specimens for biochemical analysis. *Results*. Administration of AgNPs to rats did not produce significant loss in feed intake and body weight. However, rat exposure to AgNPs caused significant alterations to levels of serum and tissue AST, ALT, and ALP. At the 100 mg/kg AgNPs exposure, rat serum and tissue AST and ALT levels were significantly decreased (*P* < 0.05). In contrast, AgNPs administration elevated (*P* < 0.05) ALP levels in rat serum and tissues. *Conclusion*. We show evidence that AgNPs administration to Wistar rats altered some biochemical parameters.

## 1. Introduction

The small sizes of the nanoparticles and large surface to volume ratio put the nanoparticles in a position for tremendous and wide applications essentially in biomedicine. Though the small sizes of engineered nanoparticles have been linked with highly desirable properties (mechanical, electrical, and chemical) for specific uses, yet these same desirable properties are also likely to be associated with unwanted biological/toxicological reactivity [1]. This raises risk concern issues on the use of nanoparticles for biomedical purposes.

Although metal nanoparticles have received increasing attention due to their widespread medical, consumer, industrial, and military applications, studies have correlated particle size of some metal-based nanoparticles (e.g., Ag, Au, and Cu) with toxicity, even if the same material is relatively inert in its bulk form [2, 3]. Indeed, there are increasing concerns about the safety of nanoparticles for human health and environment [4–6], highlighting the need for further investigations on the safety of metal nanoparticles. Moreover, data on the safety/toxicity profiles of metal nanoparticles are scarce.

Perhaps, the fears of health risks may not be completely unfounded, if the small sizes and the large surface area to volume ratio as well as the chemical reactivity and/or biological activity of the nanoparticles are to be considered. Also there are studies revealing the potential of nanoparticles to alter normal physiology by interacting with biomolecules in living cells [7–9] thereby causing adverse effects at the cellular, subcellular, and protein levels [3].

Furthermore, nanoparticles enter the human body through ingestion, inhalation, and skin contact or genitourinary tract and become deposited in vital organs such as brain, liver, or kidneys [10, 11]. Studies have shown that nanoparticles may change or damage cellular processes by passing through cellular membranes to interact with biomolecules leading to DNA and protein damage [12, 13] or cross the blood-brain barrier to cause neurotoxicity [14].

These factors underscore the urgent need for investigations aimed at establishing the influence of nanoparticles on biochemical parameters. The present study determined the effect of the oral administration of silver nanoparticle on some biochemical parameters in Wistar rats.

## 2. Materials and Methods

### 2.1. Reagents

Alanine aminotransferase (ALT), aspartate aminotransferase (AST), and alkaline phosphatase (ALP) assay kits were supplied by Randox Laboratory (Crumlin, UK). All other reagents were of analytical grade and were prepared in distilled water unless otherwise stated.

### 2.2. Experimental Animals

Male rats of Wistar strain weighing between 170 and 180 g were obtained from the University of Ibadan, Ibadan, Nigeria. Animals were housed in hygienic environment and allowed to acclimatize for a week before commencement of treatments. Animals were fed with commercial rat chows and given clean water* ad libitum*.

### 2.3. Preparation of Silver Nanoparticles (AgNPs)

The preparation and characterization of silver nanoparticles were as previously reported [15, 16]. Briefly, the AgNPs were made by adding 100 mM silver nitrate to a 1% (w/v) tannic acid solution (pH adjusted to 8 by adding 150 mM potassium carbonate) of polyvinylpyrrolidone (PVP) with stirring. The solution became pale yellow (AgNPs). The AgNPs were filtered using the 0.22 *μ*M filter and characterized by UV/Vis spectrophotometry (Biotek Epoch, USA), inductively coupled plasma optical emission spectrometry (ICP-OES, Cambridge, United Kingdom), and transmission electron microscopy (TEM, Brno, Czech Republic).

### 2.4. Animal Groupings and Treatments

Animals were randomly assigned into eight experimental groups A–H of five rats per group. Further details of animal distribution are as given.

 Group A served as control and received distilled water daily.
 Groups B, C, and D each were daily administered with 100, 1000, and 5000 mg/kg AgNPs, respectively, for 7 days.
 Groups E, F, and G each were daily administered with 100, 1000, and 5000 mg/kg AgNPs, respectively, for 14 days.
 Group H received 5000 mg/kg AgNPs daily for 21 days.

The AgNPs were diluted in distilled water. Doses were fixed based on a recent report which demonstrated that the LD_50_ for silver nanoparticles (AgNPs) was greater than 5000 mg/kg [17]. All treatments were orally administered with the aid of a cannula and animals sacrificed alternately 24 hours after cessation of treatments. The study was carried out in accordance with institutional guidelines on the handling of animals as approved for scientific research. The size range of the AgNPs used was between 8 and 20 nm.

### 2.5. Necroscopy

24 hours following cessation of last treatments, rats were sacrificed under anaesthetization in slight chloroform. Blood samples were obtained by cardiac puncture into clean centrifuge bottles and spun at 4000 ×g for 5 minutes (Heraeus Labofuge 300, Thermo Scientific, Hampshire, UK) to yield the serum. The heart, liver, and kidneys from each animal were excised and weighed immediately. Excised tissues were homogenized in ice-cold 0.25 M sucrose (1 : 5 w/v) using a Teflon homogeniser (Sigma-Aldrich Chemie GmbH, Munich, Germany). The tissue homogenates were centrifuged at 4000 ×g for 5 minutes (Heraeus Labofuge 300, Thermo Scientific, Hampshire, UK) to remove unbroken particulates. Various biochemical parameters were subsequently estimated from the serum and supernatants of tissue homogenates.

### 2.6. Biochemical Assays

The following biochemical parameters were determined in rat serum, kidney, liver, and heart using a UV/Vis spectrophotometer (Shimadzu, Kyoto, Japan) where applicable; protein concentrations in serum and tissue homogenates were estimated as described by Gornall et al. [18]. Aspartate aminotransferase (AST) was determined using commercial Randox kit [19, 20]. Alanine aminotransferase (ALT) was determined using commercial Randox kit [18]. Alkaline phosphatase (ALP) was determined using commercial Randox kit [21].

### 2.7. Data Analysis

All data were presented as the mean ± SEM. Data were subjected to statistical analysis using one-way ANOVA with GraphPad Prism 3.0 (GraphPad Software Inc., San Diego, CA). Post hoc test was performed using the Dunnett test. Mean values were considered to be statistically significant at *P* < 0.05.

## 3. Results

**Figure 1:**
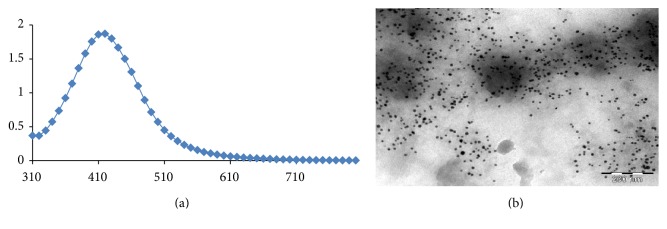
UV/Vis spectrum at 430 nm maximum (a) and TEM images of silver nanoparticles (b).

**Figure 2:**
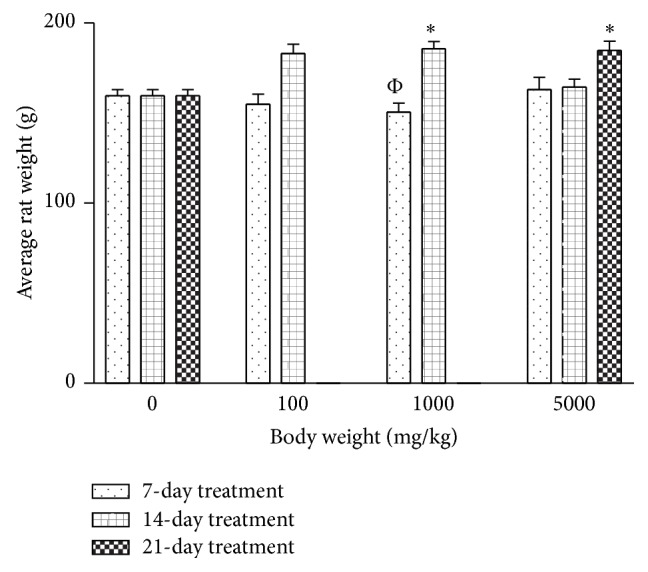
Effects of silver nanoparticles on the weight of the Wistar rats. Values are expressed as mean ± standard error of mean (SEM, *n* = 5). Values at *P* < 0.05 are significant. ∗ versus control; Φ versus 1000 mg 14-day treatment.

**Figure 3:**
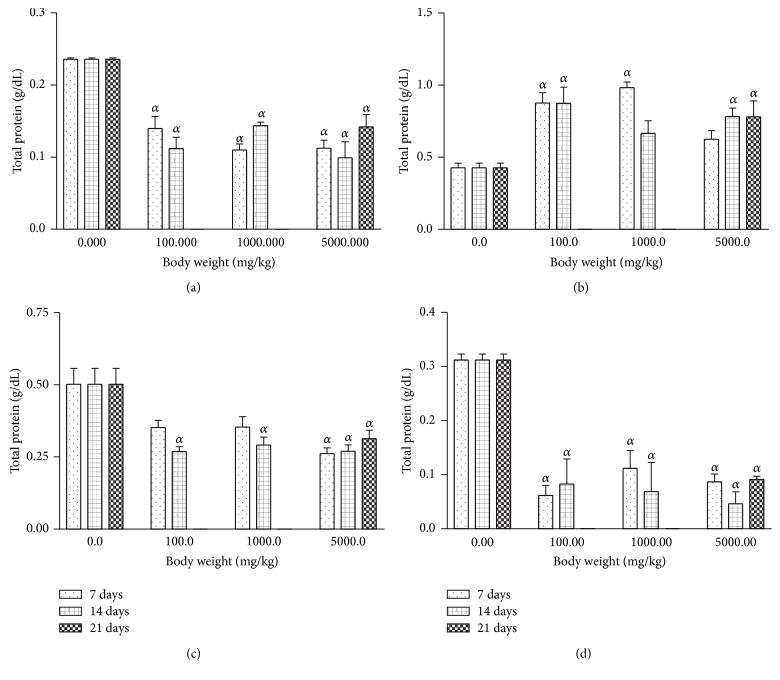
Effects of silver nanoparticles on total protein levels in rat (a) serum, (b) liver, (c) kidney, and (d) heart. Values are expressed as mean ± standard error of mean (SEM, *n* = 5). Values at *P* < 0.05 are significant. *α* versus the control group.

The AgNPs produced had a brownish yellow colour. The AgNPs had a maximal UV absorption at 430 nm with diameter sizes ranging between 8 and 20 nm (Figure 1). Treatment with AgNPs at the dose of 5000 mg/kg caused dullness in the rats, with one death recorded in the same group. No further symptoms of toxicity were observed. The relative organ-to-body weight ratios for the animals are presented in Table 1. There are no significant differences when comparing between the AgNPs treatment groups and the control. The weights of the rats treated with silver nanoparticles increased at all concentrations (Figure 2). However, the weight increments were only significant in the 1000 mg/kg for 14 days as well as the 5000 mg/kg for 21 days. Treatment of rats with AgNPs caused elevation (*P* < 0.05) in total protein levels in rat liver (Figure 3). In contrast, the levels of total protein were reduced in rat serum, kidney, and heart for groups treated with AgNPs.

**Figure 4:**
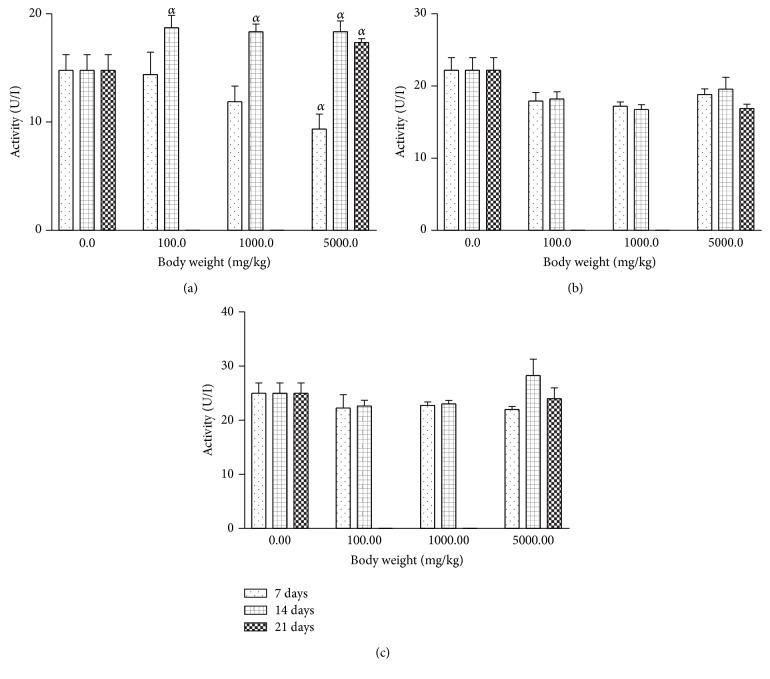
Effects of silver nanoparticles on alkaline phosphatase (ALP) levels in rat (a) serum, (b) liver, and (c) kidney. Values are expressed as mean ± standard error of mean (SEM, *n* = 5). Values at *P* < 0.05 are significant. *α* versus the control group.

Oral exposure of rats to AgNPs at various concentrations inconsistently altered the serum and tissue levels of ALP relative to the control (Figure 4). In the serum, level of ALP increased with increasing length of treatment administration. In contrast, nonsignificant reductions were recorded for tissue levels of ALP caused by AgNPs treatments.

**Figure 5:**
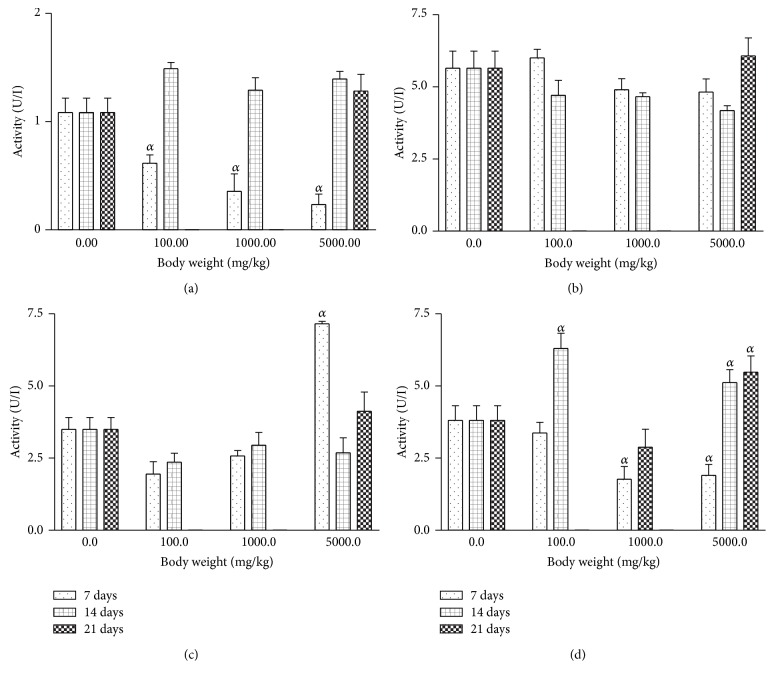
Effects of silver nanoparticles on aspartate transaminase (AST) levels in rat (a) serum, (b) liver, (c) kidney, and (d) heart. Values are expressed as mean ± standard error of mean (SEM, *n* = 5). Values at *P* < 0.05 are significant. *α* versus the control group.

**Figure 6:**
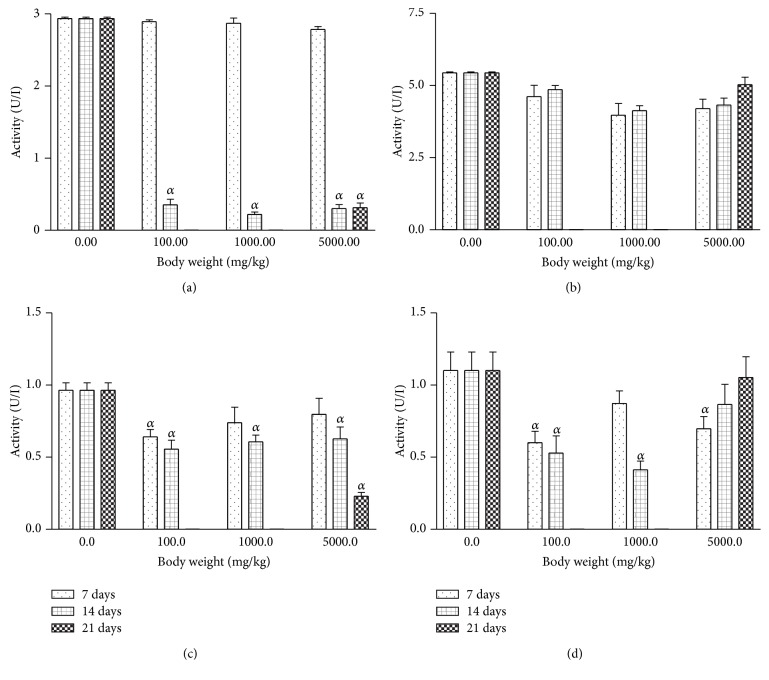
Effects of silver nanoparticles on alanine transaminase (ALT) levels in rat (a) serum, (b) liver, (c) kidney, and (d) heart. Values are expressed as mean ± standard error of mean (SEM, *n* = 5). Values at *P* < 0.05 are significant. *α* versus the control group.

For all the AgNPs treatment groups, the rat serum and tissue levels of AST were inconsistently affected relative to the control (Figure 5). Conversely, there was significant (*P* < 0.001) reduction in the levels of ALT in rat serum which was dose and duration dependent (Figure 6). On the other hand, the rat kidney and heart levels of ALT were not significantly altered by AgNPs treatment relative to control.

## 4. Discussion

The literature has shown that AgNPs are widely used due to their antimicrobial effects. Humans can be exposed to these nanomaterials via a number of routes with the nanoparticles tending to accumulate in vital organs [22]. According to Gatti [23], nanoparticle deposition in vital organs or tissues could induce cellular damage.

This study evaluated the effects of AgNPs in rats at various dosages. The AgNPs treatments did not produce significant loss in feed intake and body weight. Although one death was recorded, no further adverse signs or symptoms were recorded. The death could have been accidental. However, treatment at 5000 mg/kg AgNPs caused dullness in rats. The physical observation supports previous report [17] which reported no death and no effect on the percentage weight gain between the control and treatment groups of mice orally given 5,000 mg/kg of colloidal AgNPs.

Biochemical indices have significance in monitoring clinical symptoms produced by a toxicant. Moreover, enzyme assays play a crucial role in toxicological evaluation [24]. In the past several years, serum aminotransferases analyses have become a standard measure of hepatotoxicity because of the significance of these enzymes. Normally, these enzymes are present in the liver and other tissues where they function in energy metabolism involving the transamination of amino acids. However, in cases of cellular damage, the AST and ALT could leak out into the general circulation leading to elevated activity [24–26].

In the present study, the treatment of rats with 100 mg/kg AgNPs decreased (*P* < 0.001) levels of AST and ALT enzyme significantly, whereas there were increases (*P* < 0.001) in AST levels in serum, kidney, and heart for the 14- and 21-day treatment groups. Treatment of rats with 1000 mg/kg and 5000 mg/kg AgNPs led to a significant decrease in ALT levels and a significant increase in AST levels for the 14- and 21-day treatment groups. In contrast, AST levels significantly decreased while ALT levels increased in rat serum treated with colloidal AgNPs for 7 days when compared with those treated for 21 days [17]. This study also caused elevated (*P* < 0.05) levels of rat serum ALP levels. The effect of the oral administration of AgNPs to rats on ALP, AST, and ALT did not follow a definite pattern but may suggest partial inactivation of enzyme activity or depression of enzyme synthesis. Although the liver has been reported as one of the recurrent target organs and a dominant site of accumulation of nanoparticles [27], the alterations to the levels of enzymes may represent adaptive mechanisms by the animals trying to offset stress imposed by exposure to the AgNPs. The dosages at 100 mg/kg produced more significant alterations to the biochemical parameters than did the higher dosages at 1000 and 5000 mg/kg, respectively. However, a previous study has demonstrated that administration of AgNPs to rats caused significant alterations to the ALP levels [28]. Furthermore, alterations to the levels of AST, ALT, or ALP may not be unconnected with the affinity of AgNPs for thiol groups in protein molecules [29]. Moreover, a separate study by Srivastava et al. [30] has also demonstrated the potential of AgNPs to affect the activity of transaminase enzymes. In separate studies, the potential of silver nanoparticles to modulate enzyme activity was attributable to their affinity for thiol groups [15, 16]. It is probable that thiol groups in the enzymes made them attractive to the AgNPs leading to formation of complexes and consequent modulation of enzyme activity. Furthermore, the pronounced biochemical alterations noticed with the 100 mg/kg AgNPs in the present study may not be completely consistent with earlier report [17] in which AgNPs had an LD_50_ of >5000 mg/kg in mice.

## 5. Conclusion

The administration of AgNPs in rats did not induce major changes in body weight and feed intake. However, this study is evidence indicating that rat tissue biochemical indices were altered following oral exposure to AgNPs. Future studies are ongoing and target the determination of whether the alterations to the biochemical parameters were signs of AgNPs toxicity or not.

## Figures and Tables

**Table 1:** Effects of silver nanoparticles (AgNPs) on organ to body weight ratio of Wistar rats. Values are expressed as mean ± standard error of mean (SEM, *n* = 5).

Groups	Liver	Kidney	Heart
Control	0.033 ± 0.0034	0.0056 ± 0.00065	0.0029 ± 0.00008
100 mg/kg for 7 days	0.026 ± 0.0054	0.0055 ± 0.00016	0.0029 ± 0.00047
1000 mg/kg for 7 days	0.035 ± 0.0033	0.006 ± 0.00039	0.0038 ± 0.00023
5000 mg/kg for 7 days	0.032 ± 0.0011	0.0054 ± 0.00014	0.0032 ± 0.00023
100 mg/kg for 14 days	0.032 ± 0.0012	0.0050 ± 0.00031	0.0028 ± 0.00016
1000 mg/kg for 14 days	0.030 ± 0.0014	0.0057 ± 0.00034	0.0030 ± 0.00019
5000 mg/kg for 14 days	0.033 ± 0.0018	0.0053 ± 0.00037	0.0031 ± 0.00024
5000 mg/kg for 21 days	0.036 ± 0.0011	0.0059 ± 0.00031	0.0031 ± 0.00008
